# Nanoparticles for Cerenkov and Radioluminescent Light Enhancement for Imaging and Radiotherapy

**DOI:** 10.3390/nano10091771

**Published:** 2020-09-07

**Authors:** Federico Boschi, Antonello Enrico Spinelli

**Affiliations:** 1 Department of Computer Science, University of Verona, Strada Le Grazie 15, 37134 Verona, Italy; 2 Experimental Imaging Center, San Raffaele Scientific Institute, Via Olgettina 60, 20132 Milan, Italy; spinelli.antonello@hsr.it

**Keywords:** nanoparticles, nanocompounds, nanoclusters, cerenkov radiation, cerenkov luminescence imaging, photodynamic therapy, gold nanoparticles, silica nanoparticles, rare-earth nanoparticles

## Abstract

Cerenkov luminescence imaging and Cerenkov photodynamic therapy have been developed in recent years to exploit the Cerenkov radiation (CR) generated by radioisotopes, frequently used in Nuclear Medicine, to diagnose and fight cancer lesions. For in vivo detection, the endpoint energy of the radioisotope and, thus, the total number of the emitted Cerenkov photons, represents a very important variable and explains why, for example, ^68^Ga is better than ^18^F. However, it was also found that the scintillation process is an important mechanism for light production. Nanotechnology represents the most important field, providing nanosctructures which are able to shift the UV-blue emission into a more suitable wavelength, with reduced absorption, which is useful especially for in vivo imaging and therapy applications. Nanoparticles can be made, loaded or linked to fluorescent dyes to modify the optical properties of CR radiation. They also represent a useful platform for therapeutic agents, such as photosensitizer drugs for the production of reactive oxygen species (ROS). Generally, NPs can be spaced by CR sources; however, for in vivo imaging applications, NPs bound to or incorporating radioisotopes are the most interesting nanocomplexes thanks to their high degree of mutual colocalization and the reduced problem of false uptake detection. Moreover, the distance between the NPs and CR source is crucial for energy conversion. Here, we review the principal NPs proposed in the literature, discussing their properties and the main results obtained by the proponent experimental groups.

## 1. Introduction

Cerenkov luminescence imaging (CLI) was introduced a decade ago and is becoming an established imaging modality. Nowadays, applications include preclinical molecular imaging, surgery, external beam radiotherapy, photodynamic therapy and endoscopy. Several reviews focusing on CLI discovery and applications can be found in literature [1,2,3,4,5].

As will be described in more detail, the main problems with CLI are the low light yield and a radiation spectrum shifted towards the ultraviolet (UV)-blue region. These can become limiting factors for in vivo imaging, considering the high tissue absorption in this wavelength range [6]. In order to overcome the intrinsic limitations related to the emission of Cerenkov radiation (CR), different approaches based on the use of nanoparticles (NP) have been developed.

The main goal of this review is to provide an up-to-date overview of the use of nanoparticles as a tool to enhance the Cerenkov signal for in vivo imaging. Attention will be focused on Cerenkov light production with radioisotopes commonly used in preclinical imaging research.

This paper reviews the physics of Cerenkov light production, the applications of nanoparticles for imaging and therapy by using Cerenkov sources with a special focus on photodynamic therapy (PDT), and finally, presents a table listing the tested nanocompounds and the principal results reported in literature.

## 2. Physics of Cerenkov Light Production

**Figure 1:**
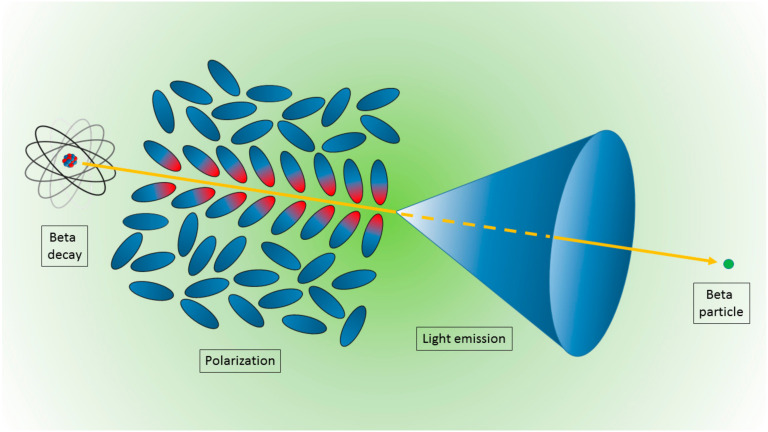
Schematic representation of the Cerenkov light emission process. A beta particle, emitted by a radionuclide, travels in a medium faster than the light speed in the medium itself. The fast de-polarization of the molecules produces a cone of light known as Cerenkov radiation.

The mechanism of CR production is rather unique with respect to other, more commonly used charged particles and matter interaction mechanisms [7]. In this case, when a charged particle (for preclinical imaging, typically a beta particle) travels through a dielectric medium, the material becomes locally polarized, with the atoms comprising the medium behaving like elementary dipoles (Figure 1). If the particle’s speed exceeds the speed of light in the medium, the polarization field becomes asymmetric along the particle track, producing a dipole field at larger distances from the track. In this case, the radiation is emitted at a characteristic angle *θ* expressed by:

(1) $$\cos\theta =\frac{1}{\beta n}$$

where $\beta =\frac{v}{c}\geq \frac{1}{n}$, *v* and *c* are the speed of the particle and of light in vacuum, respectively, and *n* is the refraction index of the material. The number *n* of the produced Cerenkov photons per unit of length *l* and wavelength λ can be obtained from the following equation:

(2) $$\frac{dN}{dld\lambda }=2\pi \alpha \frac{1}{\lambda^{2}}\left(1-\frac{1}{\beta^{2}n^{2}}\right)$$

where α is the fine structure constant. The spectrum of the emitted radiation is continuous and more intense at lower wavelengths (e.g., UV and blue region) and for *β* ≅ 1 (e.g., relativistic particles). More precisely, the spectrum shape is not dependent upon the particle energy, but particles with higher energies produce more Cerenkov photons.

As derived in [8], the measured Cerenkov spectrum in vivo is heavily dependent on the Cerenkov source power, depth, tissue properties, and overall detector efficiency with respect to the wavelength. In particular, considering the higher tissue absorption between 500 and 600 nm caused by hemoglobin absorption, in order to increase light detection in vivo, it is necessary to develop strategies to shift the CR from the UV-blue to the near infrared region (NIR).

## 3. Nanoparticles for Imaging and Therapy Using Cerenkov Sources

### 3.1. Nanoparticles

**Figure 2:**
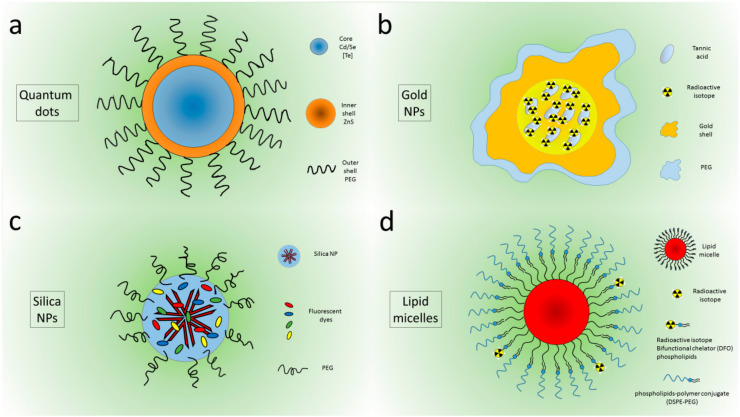
Peghilated Quantum dots (**a**), Au nanoparticles (**b**) Silica Nanoparticles (**c**), micelles (**d**).

A plethora of NPs or nanocompounds were tested to evaluate their interaction with CR sources, both for imaging and therapeutic purposes. The first attempts were made using quantum dots (QDs), because they are very bright and stable [9]. QDs are composed of a CdSe 1–6 nm-sized core, sometimes containing Te to shift the emission toward the 750–800 nm wavelength, which is useful for in vivo applications. Outside the core, their structure presents an inner shell of ZnS and an outer shell often composed of polyethylene glycol (PEG) to improve biocompatibility (Figure 2a).

They have an efficiency which is 20–30 times higher than that of organic fluorophores, size-tunable emission, an extremely broad excitation range and narrow emission which allow large Stokes shifts to occur [10]. In pioneering works, QDs were initially used as free NPs separated from the CR source [11,12,13,14]. They were then doped with ^64^Cu by Sun et al. [15] to synthetize self-illuminating NPs exhibiting high Cerenkov resonance energy transfer (CRET). QDs showed no dissociation of ^64^Cu from the NPs, and their application in in vivo tumor imaging made it possible to obtain a PET confirmation. In 2017, Zhao et al. used QDs encapsulated with ^89^Zr in lipid micelles to obtain a bimodal tracer to study pharmacokinetics and bioditribution in whole body imaging via PET and CLI [16]. Unfortunately, QDs contain toxic elements like cadmium, and thus, require external passivation for in vivo imaging applications, raising concerns about their synthesis procedures. Alternative materials with fewer safety and environmental risks are, therefore, of interest for this kind of application. For this purpose, many different NPs have been tested in the literature. Gold nanoparticles, rare earth nanophosphors, lipid nanomicelles, silica nanoparticles and titanium dioxide NPs are the most investigated compounds.

Au nanocages incorporating ^189^Au atoms were first proposed by Wang et al. in 2013, showing emission in the visible and near-infrared range and allowing the imaging of whole animals in vivo [17]. In 2014, self-illuminating gold nanoclusters doped with ^64^Cu were designed for bimodal imaging (PET and CLI) [18]. In the same year, nanostructures with a similar size but four different shapes (nanospheres, nanodisks, nanorods, and cubic nanocages) incorporating radioactive ^198^Au were investigated. The PEGylated Au nanostructures were injected intravenously in tumor-bearing mouse, and CLI was used to detect the biodistribution in vivo, confirming the results obtained with autoradiographic imaging on slices of the tumor after excision. In particular, the results showed a higher tumor uptake for the nanospheres and nanodisks compared to the nanorods and nanocages at 24 h postinjection. Interestingly, nanospheres and nanodisks were observed only on the boundaries of the cancer masses, while nanorods and nanocages were distributed throughout the tumors [19]. Gold nanoclusters conjugated with blood serum proteins were used to convert beta-decaying radioisotope energy into tissue-penetrating optical signals with both ^18^F and ^90^Y [20]. Lee et al. showed that PEGylated radioiodine-embedded gold nanostructures (Figure 2b) are promising potential lymphatic tracers in biomedical imaging for pre- and intra- operative surgical guidance [21,22].

Hollow mesoporous silica NPs and nanoshells were applied principally in photodynamic therapy (PDT); doped with ^89^Zr, they were used to activate chlorin e6 (C6) and porphyrin to produce reactive oxygen species (ROS) to damage cancer cells [23,24]. Amorphous silica NPs were tested by Pratt et al. [25] in a complete study of both NPs and CR sources. Recently, silica NPs were synthesized containing five different dyes that were chosen to efficiently absorb CR in a wide wavelength range and to efficiently tunnel the excitation energy toward the lowest energy Cy7 derivative dye presenting a fluorescence emission in the near-infrared region (NIR), [26] (Figure 2c).

Lipid calcium phosphate nanoparticles bound with ^177^Lu were investigated by Satterlee et al. [27] to perform both anticancer therapy and in vivo imaging, exploiting CLI and SPECT and imaging modalities. Other liposomes containing ^188^Re were developed by Chang et al. [28] to reduce proliferation of human and neck cancer cells in vivo. Lipid micelles doped with ^89^Zr and containing QDs were investigated by Zhao et al. [16] to image pharmacokinetics and biodistribution using both PET and CLI techniques (Figure 2d).

TiO_2_ NPs were investigated almost exclusively for PDT, as shown in the next section. The use of rare earth nanophosphors was suggested in 2011 by Sun et al. [29]. Carpenter et al. [30] demonstrated the down-conversion of Cerenkov light emitted from ^18^F by barium yttrium fluoride nanoscrystals doped with terbium and europium. Instead, excitation by ^68^Ga terbium-doped Gd nanoparticles revealed a significant improvement in detection sensitivity for clinical diagnoses of gastrointestinal tract tumors [31]. The fact that gamma radiation is the major cause of Europium Oxide (EO) NP emissions was demonstrated by Hu et al. [32]. The same group successfully employed EO NPs in cancer detection, even with ultrasmall tumors (less than 1 mm) [33,34].

A great variety of NPs (silica, TiO_2_, rare earth, gold NPs) were tested by Pratt et al., who investigated both sources and NPs in order to clarify the excitation mechanisms [25] underlying the NP light emission.

Recently, persistent luminescence nanoparticles (PLNPs) with rechargeable near infrared afterglow properties, specifically, Cr^3+^-doped zinc gallate (ZGCs), were proposed in combination with ^18^F for tumor diagnosis in living animals, since they can avoid tissue autofluorescence and improve the signal-to-background ratio [35].

### 3.2. Toxicity

In order to evaluate the effects of NPs on living organisms, their toxicity has been investigated in many different modalities. It is worth noting that for diagnostic purposes, safe NPs are requested, as opposed to therapeutic applications, whereby specific cytotoxicity against cancer cells (combined with a high degree of specific uptake) could represent an advantage. In particular, for PDT, NPs need to exhibit low cytotoxicity in the dark and high killing when activated by light. Black et al. [36] evaluated the toxicity of cationic block copolymer NPs in HEK293T cells; the viability was determined by measuring the ATP activity. Lee et al. [21] examined the cytotoxicity of gold NPs on various cell types, including Chinese hamster ovary (CHO) cells, murine macrophage (Raw 264.7) cells, and mouse dendritic (DC2.4 cells) cells using cell viability and apoptosis assays. The results were supported by flow cytometry analysis following annexin V and propidium iodide staining, suggesting that PEG-RIe-AuNPs are not toxic to normal ovarian or immune cells. Besides flow cytometry for in vitro assays, to evaluate the tissue toxicity of EO nanoparticles, Hu et al. [34] examined different organs extracted from human breast (Bcap-37) xenograft mice stained with hematoxylin and eosin (H&E). Goel et al. [24] evaluated the effects of hollow mesoporous silica NPs on white blood cells, red blood cells, mean corpuscular volume, hemoglobin, hematocrit, mean corpuscular hemoglobin, and platelet, collected in mice injected with therapeutic doses of mesoporous silica nanoshells filled with porphyrin at different timepoints after injection. Duan et al. [37] reported the cytotoxicity of the D-TiO_2_ NPs evaluated by Cell Counting Kit CCK8 assay. Lee et al. [38] tested PEGylated crushed gold shell-radioactive iodide-124-labeled gold core-124I nanoballs (PEG-Au@AuCBs) with a Cell Counting Kit CCK8 and a CellTiter-Gl Luminescent Cell Viability Assay. Chang et al. [28] used histochemical measurements of stained Ki-67 expression in orthotopic tumors to evaluate the toxicity caused by single and repeated doses of 188Re-liposomal administrated in tumor-bearing mice.

### 3.3. Radioisotope/Nanoparticle Interaction

Initially, the link between NPs and CR sources was thought of as a simple interaction between Cerenkov photons and fluorescent systems. It subsequently emerged that the beta plus and beta minus particles produced in the decay also interact with the fluorescent systems, releasing the energy needed to emit new photons. For some NPs, gamma emission shows a very low efficiency, and in general, can be ignored [20].

**Figure 3:**
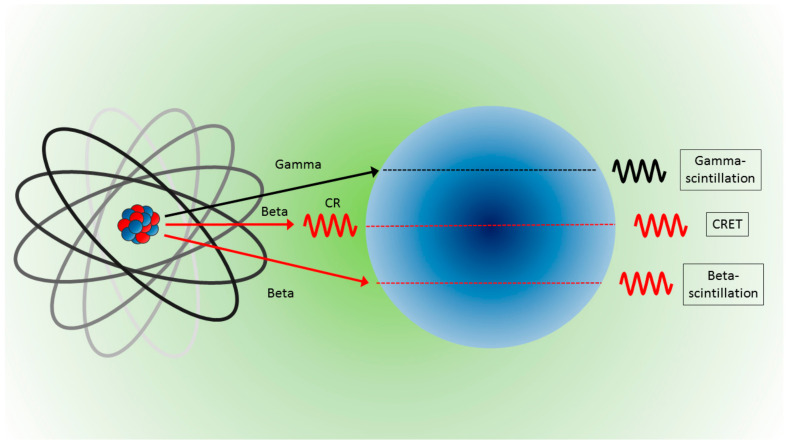
Light produced from NPs and the interaction with radionuclides are due to gamma rays which produce gamma scintillation (**top**), interactions with photons (Cerenkov radiation) which are converted by CRET (**middle**), and direct interactions with beta particles which produce beta scintillation (**bottom**).

More precisely, radionuclides and NPs can interact in various ways to produce light. NPs can convert the energy released from gamma radiation (gamma scintillation) or beta particle (beta scintillation) into visible photons, or NPs can be excited from photons produced by beta particles (Cerenkov radiation) and deexcited to emit visible photons (with longer wavelengths) via the so-called Cerenkov radiation energy transfer (CRET). A schematic of the possible interaction is illustrated in Figure 3. In any case, considering the form of energy reaching and emitted by the NPs, interactions can be categorized into two processes: photon–photon and beta–photon emission. For a detailed description of the physical processes behind each kind of interaction, see the work of Pratt et al. [25] and the complete review of Ferreira et al. [39].

### 3.4. Optical and Chemical Properties

For imaging purposes, the best NP candidates need to have specific optical properties: absorption spectrum compatible with CR emission, high quantum efficiency and emission in the wavelength range 650–800 nm. This wavelength range of lower optical absorption coefficient is known as tissue transparency window. In cases of excitation via optical photons, the distance between the CR source and the NPs could represent an issue, requiring that NP absorption spectrum be compatible with the red-shifted Cerenkov radiation in the tissue, and that the wavelength of the incoming photons themselves be in the tissue transparency window. In general, the distance, and consequently, the chemical link between the CR source and the NPs is a very important aspect. Nps separated from radionuclides normally have different biodistribution, presenting a low degree of colocalization and reduced CR conversion efficiency.

Separated NPs need to be functionalized in order to reach the same target (generally a tumor mass) as the radionuclide. A simpler strategy is to bind radionuclides and NPs, or encapsulate them therein, or substitute the chemical components of the NPs with radioactive isotopes. Following the review of Shaffer et al. [5], NPs were subdivided in into three groups: NPs not linked to the CR sources (separated), NPs bound to the emitters (bound) and NPs incorporating the CR sources (incorporated), often called “self-emitting NPs”. The chemical link must be stable in vivo to prevent false localization via imaging techniques, but not all the chemical links are stable or easy to obtain, and thus, the biodistribution of the complex CR source-nanoparticles could be different from that of the NPs. NPs bound and incorporating radioisotopes are a good solution to the problem of excitation distance and colocalization; however, the need to control specific uptake in the target organ remains.

### 3.5. Applications to Cancer Models

Principal biomedical applications are whole body imaging for preclinical investigations, especially for tumor and Lymph nodes detection, endoscopy, and PDT. The most used cell lines for in vivo cancer models are 4T1 murine breast cancer [23,24,32,37,40,41] murine mammary carcinoma EMT-6 [17,19], human primary glioblastoma U87 [15,18,32], human fibrosarcoma HT 1080 [42,43,44], and human hepatocellular carcinomas HCC [33,34]. The 4T1 murine breast cancer model has been employed mostly for in photodynamic therapy studies [23,24,37,41]. It is worth nothing that breast cell lines are very commonly used for testing due to the shallow nature of the associated tumor model, and thus, its in vivo detectability, and for the clinical translatability of the results.

### 3.6. CR Sources

Initially, the most widely used CR sources were ^18^F and ^64^Cu for direct involvement in clinical and preclinical investigations with PET [12], and in particular, ^18^F for the widespread use of 18F-FDG in cancer detection. Subsequently, also ^32^P and ^68^Ga [13,14] were investigated for their higher production of Cerenkov photons due to their higher endpoint energy, leading to improve in vivo detectability. As mentioned, the choice of the radioisotope is important, not only for its optical emission (related to the activity and to the endpoint energy), but also for its decay mode, which can produce particles which are able to interact with NPs, causing light emission. The principal radioactive sources tested by different authors are ^3^H, ^35^S, ^177^Lu, ^32^P, ^18^F, ^89^Zr, ^68^Ga, ^90^Y and ^99m^Tc, [25].

Considering its therapeutic application in the FDA-approved treatment of patients with gastro-entero-pancreatic neuroendocrine tumors [45], lutetium was investigated as a CR source by Satterlee et al. [27], bound to lipid calcium phosphate NPs—as reported above regarding the lipid nanostructures—as a mode of anticancer therapy in addition to radiographic imaging. Analogously, ^188^Re, normally used for bone pain palliation in patients suffering prostate cancer [46], was encapsulated in lisosomes by Chang et al. [28] to inhibit proliferation and epithelial-mesenchymal transition of human and neck tumors.

### 3.7. Imaging Techniques

PET imaging is a widely used alternative technique to detect radionuclide-NP complexes. However, SPECT was used by Lee et al. [47], who imaged ^125^I-radiolabeled gold nanoparticles. Superparamagnetic iron oxide nanoparticles labeled with ^124^I or ^18^F-FDG were investigated by [42] to achieve MRI detection of the complex, also in triple optical/PET/MRI modality [40]. It was also reported that the shape-controlled synthesis of rare earth fluoride nanocrystals doped with the β-emitting radioisotope ^90^Y may provide a promising platform for multimodal imaging, including MRI [48].

## 4. Photodynamic Therapy Using Cerenkov Sources

The use of light to treat diseases can be dated back to antiquity, as outlined in these two reviews [49,50] focusing on the history of PDT. For example, the ancient Greek physicians, and Herodotus (the father of heliotherapy) in particular, suggested the use of sunlight to treat diseases.

Modern PDT can be traced back to the early 1900s, when the Danish physician, Niels Finsen treated respectively with red or UV light pustules or cutaneous tuberculosis, and was awarded the Nobel Prize in 1903 for his efforts. Around that time, the idea of using light in combination with a drug to kill cells was suggested by Oscar Raab, a medical student who, during a thunderstorm, discovered (by chance) the lethal combination of acridine and light on Infusoria. His supervisor Herman von Tappeiner went on to show the importance of oxygen in the reaction, and in 1907, he introduced the term “photodynamic action”. The basic mechanisms of PDT were thus discovered.

**Figure 4:**
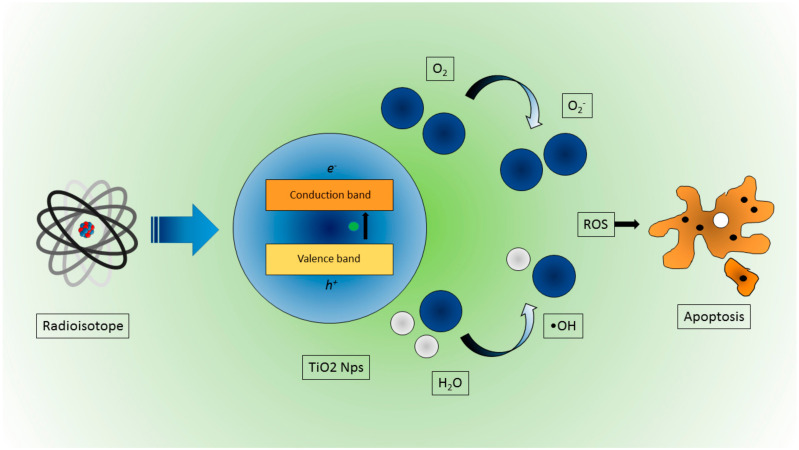
Schematic representation of ROS production due to CR interaction in TiO_2_ NPs, leading to cancer cell apoptosis.

More precisely, PDT is based on the interaction between light at a specific wavelength and a photosensitizer (PS) that is a photo-activatable molecule (at a given wavelength). After the interaction with light, the PS is able to produce reactive oxygen species (ROS), leading to local cytotoxic reactions with cells (Figure 4). Of course, in order to achieve a significant cytotoxic effect, both light and the PS should available where needed (e.g., inside a tumor region), avoiding normal tissue at the same time. It is thus necessary to develop targeting strategies to enhance the selectivity of the PS and, at the same time, to radiate enough light into the region that needs to be treated. This latter requirement limits the use of PDT, since, due to hemoglobin absorption and tissue scattering, it is not a trivial matter to deliver enough photons to nonsuperficial regions.

In order to solve this problem, there has been, in the recent years, a growing interest in using CR as a local and internal light source, or other external excitation sources like x-rays. The main advantages of using Cerenkov sources are the possibility of bringing the light source as close as possible to the PS and using the UV component of the CR spectrum. This approach was applied by Kotagiri et al. [43]; specifically, transferrin-coated TiO_2_ nanoparticles in combination with PET radionuclides like ^18^F and ^64^Cu were used to perform PDT in vitro and in vivo. They found a strong reduction of cell viability in vitro with respect to control groups when combining radionuclides with the PS. A similar trend was found in vivo using an aggressive HT1080 subcutaneous mouse tumor model. In this case, the tumor volume dropped to almost zero 12 days after treatment, while the controls groups were in the 400–600 mm^3^ range. A histological analysis also confirmed extensive tumor necrotic regions.

As mentioned, the number of Cerenkov photons that are produced is dependent on the particle energy. For PDT, it is thus useful to choose, when possible, beta emitter isotopes with higher end point energies in order to obtain a higher yield of Cerenkov photons [51]. Instead of ^18^F, Duan et al. [37] suggested the use of ^68^Ga (end point energy = 1899 keV), while Hartl et al. [52] proposed the use of ^90^Y (end point energy = 2.28 MeV) as an even brighter PDT source with a longer half-life.

Different combinations of radioisotopes and PSs have been investigated, for example in Kamkaew et al. [23]. ^89^Zr was used as a Cerenkov PDT source to excite chlorin e6 (Ce6), yielding similar in vitro and in vivo results (e.g., lower cell viability and tumor volume) by Kotagiri at al. [43].

In a commentary paper [53], the authors outlined the hypothesis that due to the low yield of Cerenkov emission, an alternative OH radical production mechanism should be sought. It has thus been suggested that an increase in free radical production might be achieved by interactions between direct beta particles and the PS. However, further evidence [53] using ionizing radiation below the Cerenkov threshold showed very little difference in cell viability between groups irradiated with the PS and those without, suggesting the importance of Cerenkov light as a PDT source.

The mechanisms responsible for the creation of electron/hole pairs in the PS need to be further investigated in order to better understand the contribution of possible sources.

## 5. Quick Overview

A table containing a list of the most interesting NPs used in combination with radionuclides is presented in this Section (Table 1). The list is focused on biomedical applications, i.e., mainly imaging and phototherapy. Some other contributions were added due to their interesting results, despite the aim of the research being different from biomedical applications.

## 6. Discussion and Conclusions

Since the discovery of Cerenkov luminescence imaging, much effort has been made to overcome the small penetration depth of CR in soft tissue. Nanotechnology represents the most important field, providing nanosctructures which are able to shift UV-blue emissions into more suitable wavelengths, with reduced absorption, which is especially useful for in vivo imaging applications and for therapy. A large variety of NPs with different optical/chemical properties have been tested to date. Nanoparticles can be made, loaded or linked to fluorescent dyes to modify the optical properties of CR radiation, but they also represent a useful platform for therapeutic agents, i.e., as photosensitizer drugs. They can also be categorized according to the CR source; however, for in vivo imaging applications, NPs bound to or incorporating radioisotopes are the most interesting nanocomplexes thanks to their high degree of mutual colocalization and the reduced problem of false uptake detection. Moreover, the distance between the NPs and CR source is crucial for energy conversion.

For in vivo detection, the endpoint energy of the radioisotope and, thus, the total number of the emitted Cerenkov photons, represents a very important variable and explains why, for example, ^68^ Ga is better than ^18^F. However, it was found that the scintillation process is also an important mechanism for light production, while gamma radiation seems to contribute less to the overall luminescence. Finally, as shown above, the toxicity of NPs has been tested by different groups in many alternative modalities, but without a comparison of their effect on cells and organs; therefore, a more uniform procedure could help in assessments of their toxicological properties.

An interesting approach to improving signal-to-noise ratios could be the use of time-gated radioluminescence using a CCD camera synchronized with the X-ray radiation pulse [65] or silicon nanocrystals with long emission lifetimes [66].

On the therapeutic side, the development of Cerenkov sources along with external x-ray sources will play an important role in overcoming the limits of light penetration for PDT [67,68,69].

Overall, in the coming years, we will likely witness improvements of the NPs which have already been tested and the synthesis of new NPs with better optical and chemical properties, aiming to increase the specific uptake and reduce the toxicological risk.

## Figures and Tables

**Table 1:** List of studies reporting the interactions between Cerenkov sources and nanosized particles. Briefly, the information reported in the columns is: progressive number (1), nanoparticles tested (2), Cerenkov radiation used to excite the nanoparticles (3), type of link between CR source and NPs (4), application of the study (mainly in vitro, in vivo, ex vivo) and cell lines in case of cancer imaging/therapy (5), main results of the study (6), notes (7), optical instrument used (8), reference (9). For an exhaustive explanation, see the main text.

N	NPs	Source	NP–CR Source Link	Applications	Main Results	Note	Instrument	Reference
01	CdSe/ZnS Quantum dots QD655, QD705, QD800	131 I	Separated	In vitro and in vivo.	Feasibility of using radiation luminescence as an internal source to illuminate QDs.		IVIS 200 and IVIS Spectrum	Liu et al. (2010) [11]
02	Quantum dots (Qtracker705)	64Cu, 18F and 99mTc	Separated	In vitro and in vivo.	Qtracker705 and 18F-FDG showed CRET ratios in vitro as high as 8.8 ± 1.1. In vivo in pseudo tumor impregnated with Qtracker705 following intravenous injection of 18F-FDG showed CRET ratios as high as 3.5 ± 0.3. No efficient energy transfer detected with 99mTc.	Definition of Cerenkov radiation energy transfer (CRET) ratio.	IVIS 100	Dothager et al. (2010) [12]
03	Tyramine conjugated superparamagnetic iron oxide nanoparticle (TCL-SPION) C = 4–11 nm H ~40 nm	124I	Bound	In vitro and in vivo. Sentinel lymph nodes (SLNs) detection of mouse bearing breast 4T1 tumor.	Facilitated noninvasive differentiation between tumor-metastasized sentinel lymph nodes (SLNs) and tumor-free SLNs	Triple-modality optical/PET/MRI.	IVIS 200	Park et al. (2010) [40]
04	Radioluminescent nanophosphors (RLNPs) Barium yttrium fluoride (Ba0.55Y0.3F2) doped with EuropiumC = 14 nm	18F	Separated	In vitro and in vivo.	Presentation of facile synthesis and surface modification process to produce water-soluble radioluminescent lanthanide-doped nanophosphors. FDG-stimulated optical imaging of the mice bearing inclusions clearly displayed enhanced emission at 700 nm on the flank containing RLNPs	PET and X-ray validation	IVIS Spectrum	Sun et al. (2011) [29]
05	Radioluminescent nanophosphors (RLNPs) Barium yttrium fluoride (Ba0.55Y0.3F2) nanocrystals doped with terbium (0.5%) or europium (0.5%) C = 14 nm H ~27 nm	18F and X-ray tube	Separated	In vitro and in vivo.	RLNPs doped with terbium or europium can be distinguished in optical images in gelatin phantoms. RLNPs aid in the down-conversion of Cerenkov light emitted from the radiopharmaceutical.	PET validation.	IVIS Spectrum	Carpenter et al. (2012) [30]
06	Quantum dots (QD800)	32P	Separated	In vitro.	Primary CR and beta particles contribute almost equally to the excitation of the QDs. Good agreement of the light intensity emission with the inverse squared law of the NP–CR source distance.	P32 source covered alternatively with plexiglass slabs or black paper to obtain pure Cerenkov source or pure beta emitter source	IVIS Spectrum	Boschi et al. (2012) [13]
07	Au nanocages C = 33 nm	198Au	Incorporated	In vitro, in vivo, ex vivo. murine mammary carcinoma (EMT-6)	Au nanocages show emission with wavelengths in the visible and near-infrared regions, enabling luminescence imaging of the whole mouse in vivo, as well as the organs ex vivo.	First incorporation of CR source into the nanostructure for imaging purpose	IVIS Lumina II XR	Wang et al. (2013) [17]
08	Fluorescenin (FAM/FITC). Cyanine (Cy5.5, Cy7 ICG). Quantum dots (QD565, QD605, QD800). Au NPs	68Ga, 18F, 89Zr	Separated	In vitro, in vivo. Mouse squamous cell carcinoma (SCC-7) and human breast cancer (BT-20).	Reduced background signal compared to conventional fluorescence imaging. Approach useful to quantitatively determine prognostically relevant enzymatic activity.	PET and CT validation	IVIS200	Thorek et al. (2013) [14]
09	Gold nanocluster C = 2.56 ± 0.50 nm	64Cu	Bound	In vitro, in vivo, ex vivo. Human primary glioblastoma (U87).	Portion of the energy of Cerenkov radiation serves to excite AuNCs in 64Cu-doped AuNCs. 64Cu-doped AuNCs can be applied as an alternative indicator of PET signal.	Self-illuminating gold nanocluster for dual-modality PET and near-infrared (NIR) fluorescence imaging	IVIS Lumina II	Hu et al. (2014) [18]
10	Quantum dots (CdSe/ZnS QDs) QD526 QD580 QD636 H = from 14.1 to 28.4 nm PEG-coated	64Cu	Bound	In vitro, in vivo Human primary glioblastoma (U87).	Favorable imaging without the issue of dissociation of 64Cu from the particles and controllable and enhanced long-wavelength luminescence emission detectable by in vivo imaging.	First time for direct doping of 64Cu PET isotope into QDs via a cation-exchange reaction and endowing them with luminescence properties. PET validation	IVIS Lumina II	Sun et al. (2014) [15]
11	Au nanostructures: nanospheres, nanodisks, nanorods, nanocages	198Au	Incorporated	In vitro, in vivo, ex vivo. Murine mammary carcinoma (EMT-6)	Nanospheres showed the best blood circulation, the lowest clearance by the reticuloendothelial system, and the highest overall tumor uptake relative to nanodisks, nanorods, and nanocages. Nanorods and nanocages could reach the cores of the tumors, whereas nanospheres and nanodisks were only observed on the surfaces	PET and autoradiography validation	IVIS 100	Black et al. (2014) [19]
12	Superparamagnetic iron oxide nanoparticles (SPIO)	18F	Separated	In vitro, in vivo. Human fibrosarcoma (HT1080)	Demonstration of quenching of Cerenkov emissions using nanoparticle platforms to provide disease-relevant information including tumor vascularity and specific antigen expression	Several proof of principle models using nanoparticles and clinically approved agents PET validation	IVIS 200	Thorek et al. (2014) [42]
13	(PEG)-coated TiO_2_ nanoparticles, transferrin-coated TiO_2_ nanoparticles titanocene-transferrin- TiO_2_ nanoparticles	18F, 64Cu	Separated	In vitro, in vivo, ex-vivo Human fibrosarcoma (HT1080)	Observed a remarkable shrinkage of the tumor volume (40 ± 5%) within three days of CR-induced therapy initiation. Complete tumor regression was achieved by 30 days and translated into complete remission without a significant loss in body weight up to four months posttreatment	Phototherapy Fluorescence imaging and PET validation		Kotagiri et al. (2015) [43]
14	Si nanoparticles	Deuterium lamp	Separated	Not for bioimaging applications. New sensor materials field.	Placing a film of nanoparticles in front of a standard visible-wavelength detecting photosensor, the response of the sensor was significantly enhanced at wavelengths < 320 nm.	To enhance Cerenkov emission and for all experiments requiring sensitivity to UV photons	Hamamatsu MPPC	Magill et al. (2015) [54]
15	Terbium doped Gd_2_O_2_S (Gd_2_O_2_S:Tb)nanoparticles	68Ga	Separated	In vitro, in vivo Gastro intestinal tumor	50-fold improvement in detection sensitivity, which guaranteed meeting the demands of the clinical diagnosis of gastrointestinal tract tumors.	Endoscopy	EMCCD camera iXon3 888, Andor	Cao et al. (2015) [31]
16	Terbium doped Gd_2_O_2_S Microparticles C = few µm	18F	Separated	In vitro, in vivo Gastro intestinal tumor	RLMPs significantly improve the intensity and the penetration capacity of CLI, which has been extended to as deep as 15 mm. Microparticles can be excited by gamma rays, but can barely be excited by Cerenkov luminescence.		IVIS system	Cao et al. (2015) [55]
17	Europium oxide nanoparticles (EO) C = 85 ± 22 nm.	18F, 99mTc, 131I	Separated	In vitro, in vivo, ex vivo human breast tumor (Bcap-37) mouse breast tumor (4T1) human glioma tumor (U87MG) human liver tumor (HepG2)	Gamma radiation is the major cause of EO excitation. Strong optical signals with high signal-to-background ratios, an ideal tissue penetration spectrum and activatable imaging ability. More effective detection of tumor lesions with low radioactive tracer uptake or small tumor lesions.	Comparison with Quantum dots (QD620) PET validation	IVIS system	Hu et al. (2015) [32]
18	Gold nanoclusters conjugated with blood serum proteins (AuNCs)	18F, 90Y, 99mTc	Separated	In vitro, in vivo, ex-vivo Breast carcinoma (MDA-MB-231)	AuNCs convert beta-decaying radioisotope energy into tissue-penetrating optical signals between 620 and 800 nm with 18F and 90 Y but not with 99mTc. Optical emission from AuNCs is not proportional to Cerenkov radiation, indicating that the energy transfer between the radionuclide and AuNC is only partially mediated by Cerenkov photons. Excitation by high-energy photons is highly inefficient.	Definition of luminescence output (LO) of AuNC as a result of interactions with radioisotope	IVIS Spectrum	Volotskova et al. (2015) [20]
19	GdF3:90Y/Y nanoplates C = from 8.1 ± 1.2 nm to 15.5 ± 1.3 nm	90Y	Incorporated	In vitro	Synthesis of a plethora of nanocrystals with different shapes doped with 90Y. Linear relationship between total radiance and radioactivity, suggesting that 90Y-doped nanocrystals are applicable for quantitative optical imaging studies.	Evaluation of MRI capabilities of 90Y-doped GdF3 nanocrystals	IVIS Lumina II	Paik et al. (2015) [48]
20	Lipid-calcium-phosphate nanoparticle (177Lu-LCP) H = 36 ± 9 nm	177Lu	Bound	In vitro, in vivo, ex vivo Human nonsmall cell lung cancer cells (H460) Human bladder cancer cells (UMUC3)	177Lu-LCP functioned as in vivo anticancer therapy in addition to radiographic imaging via the dual decay modes of 177Lu. Treatment with just one dose of 177Lu-LCP showed significant in vivo tumor inhibition in two subcutaneous xenograft tumor models.	Tumor accumulation of 177Lu-LCP was measured using both SPECT and Cerenkov imaging modalities in live mice.	IVIS Kinetic	Satterlee et al. (2015) [27]
21	Glucose-based polymer dextran (89Zr-PNP) C = from 34 to 82 nm	89Zr	Bound	In vitro, in vivo	89Zr-PNP guided the surgical resection of sentinel lymph nodes, utilizing their Cerenkov luminescence. PNP also made it possible to monitor drug release via MRI, through the quenching of the gadolinium signal by the coloaded drug, making them a new multifunctional theranostic nanotechnology platform.	PET/CT validation	IVIS Spectrum	Kaittanis et al. (2015) [56]
22	Gd_2_O_2_S: Eu^3+^ nanophosphors	89Zr	Bound	In vitro, in vivo, ex vivo	Excitation of Gd_2_O_2_S:Eu nanoparticles by 89Zr was successfully observed. Increasing the nanoparticle concentration or radioactivity increased the intensity of the emission signals. The distance between the donor and the receptor significantly influenced the RL intensity.	PET validation	IVIS Spectrum	Ai et al. (2016) [57]
23	Titanium dioxide (titania) nanoparticles (NPs) C = 5 nm	X-ray external beam	Separated	In vitro Human lung carcinoma cells (A549)	6 MV radiation produced the most CR per unit dose deposition, i.e., about 10 times higher than that of 18F. Synergistic effect for the combination of ionizing radiation and titania NPs was observed in the 6MV experiments, where 20% more cancer cells were killed in the group with both radiation and NPs compared to with radiation alone.	External beam radiotherapy Monte Carlo simulations with 18F, 192Ir and 60Co as internal sources		Ouyang et al. (2016) [58]
24	Poly(acrylamidoethylamine)-b-poly (DL-lactide) block copolymer-based degradable, cationic, shell-cross-linked knedel-like NPs (Dg-cSCKs)H = 135 ± 40 nm	123I, 124I, 131I, 76Br	Bound	In vitro, in vivo, ex vivo.	In vivo characterization of pharmacokinetics and fate of the NPs by radiolabeling Dg-cSCKs using a multimodal, noninvasive imaging approach that incorporated positron emission tomography (PET) and Cerenkov luminescence imaging.	Intratracheal injection for lung gene transfer. PET validation	IVIS 100	Black et al. (2016) [36]
25	Radioiodine embedded gold (Au) nanoparticles (Rie AuNPs) C = 5, 20 and 42 nm	124I, 125I	Bound	In vitro, in vivo, ex vivo.	Simple and straightforward synthetic scheme for producing gold-based imaging agents that are applicable as a dual bio-imaging modality by combining nuclear imaging and CLI.	PET/SPECT validation	IVIS Lumina III	Lee et al. (2016) [47]
26	Peghilated radioiodine embedded gold (Au) nanoparticles (PEG-RIe-AuNPs) C = 20 nm	124I	Bound	In vitro, in vivo, ex vivo.	In vivo imaging reveals sentinel lymph nodes as early as 1 h post PEG-RIe-AuNP-injection, with peak signals achieved at 6 h postinjection. The data provide strong evidence that PEG-RIe-AuNPs are promising as potential lymphatic tracers in biomedical imaging for pre- and intra- operative surgical guidance	NPs useful for sentinel lymph node detection via PET and CLI PET/CT validation	IVIS Lumina III	Lee et al. (2016) [21]
27	Hollow mesoporous silica nanoparticles ([89Zr]HMSN-Ce6) H ∼130 ± 2.1 nm	89Zr	Bound	In vitro, in vivo, ex vivo. Murine breast cancer (4T1)	In vitro cell viability experiments demonstrated dose-dependent cell deconstruction as a function of the concentration of Ce6 and 89Zr. In vivo studies showed inhibition of tumor growth when mice were subcutaneously injected with [89Zr]HMSN-Ce6, and histological analysis of the tumor section showed damage to tumor tissues, implying that reactive oxygen species mediated the destruction.	Photodynamic Therapy Activation of chlorin e6 (Ce6) to generate reactive oxygen species (ROS) PET imaging	IVIS Spectrum	Kamkaew et al. (2016) [23]
28	Europium oxide (EO) nanoparticles C = 85 ± 22 nm.	131I, 18F, 68Ga, 99mTc	Separated	In vitro, in vivo, ex vivo. Human hepatocellular carcinomas (HCC).	A mixture of 68GaCl3 and EO nanoparticles yielded the strongest optical signals compared with the other mixtures. Radiopharmaceutical-excited fluorescence tomography (REFT) can detect more tumors than small-Animal PET in hepatocellular carcinoma-bearing mice, and achieved more accurate 3D distribution information than Cerenkov luminescence tomography.	PET/CT imaging.	IVIS Kinetic	Hu et al. (2017) [33]
29	Dual-labeled nanoparticles based on lipid micelles (89Zr-QD-MC) nanoemulsions (89Zr-QD-NE), and polymeric biocompatible nanoplatforms (89Zr-QD-BP). H = from 45 to 75 nm	89Zr	Bound	In vitro, in vivo, ex vivo. Human prostate cancer (DU145)	The intensity of converted light is linearly related to the concentration of the spectral converter, and the slope is related to the quantum yield of the fluorophore. Pharmacokinetics, biodistribution, and whole-body imaging of QD and 89Zr dual-labeled nanoparticles.	PET/CT imaging	IVIS Spectrum	Zhao et al. (2017) [16]
30	Europium oxide (EO) nanoparticles C = 85 ± 22 nm.	18F, 11C	Separated	In vitro, in vivo, ex vivo. Mouse breast cancer (4T1) Human hepatocellular carcinoma (HCC).	By mixing the 18F–FDG and EO nanoparticles, strong near-infrared fluorescent light is emitted, and its peak is 620 nm. The total flux is almost 70 times higher than the sum of the optical signals of EO nanoparticles and 18F–FDG alone. EO at a very low dose can be excited by radiopharmaceuticals of very low dose to produce an optical signal. Mediated radiopharmaceutical-excited fluorescent (REF) image-guided cancer surgery strategy technique exhibited excellent performance in detecting invisible ultrasmall tumors (even less than 1 mm) and residual tumor tissue.	For precise image-guided tumor-removal surgery. Employs the internal dual excitation of EO nanoparticles by both gamma rays and Cerenkov luminescence of radiopharmaceuticals.	IVIS Kinetic	Hu et al. (2017) [34]
31	Citrate-capped (Cit) copper sulfide (CuS) nanoparticles on the surface of [89Zr]-labeled hollow mesoporous silica nanoshells (HMSN) filled with porphyrin molecules H~150 nm (HMSN), ~10 nm (CuS-Cit)	89Zr	Bound	In vitro, in vivo, ex vivo. Murine breast cancer (4T1).	Development of a novel, biocompatible, hybrid nanoplatform to seek and treat cancer in vivo. [89Zr]-labeled HMSN shell, CuS nanosatellites and photosensitizer porphyrin, self-assemble for Tetramodal Imaging and Synergistic Photothermal/Photodynamic Therapy Localized and synergistic phototherapy shows complete tumor eradication with no recurrence or long-term toxicity.	Photodynamic therapy PET, fluorescence, Cerenkov Luminescence and Cerenkov Radiation Energy Transfer-based imaging, and, photothermal/photodynamic therapy Activation of TCPP and doxorubicin	IVIS Spectrum	Goel et al. (2018) [24]
32	68Ga-labeled bovine serum albumin (68Ga-BSA) and dextran-modified TiO_2_ nanoparticles (D-TiO_2_ NPs) H = from 73.2 to 83.2 nm	68Ga, 18F	Separated	In vitro, in vivo, ex vivo. Murine breast cancer (4T1)	68Ga is a more potent radionuclide than 18F for CR-induced PDT. The tumor volumes in mice treated by 68Ga-BSA and D-TiO2 NPs were significantly inhibited, whereas no significant difference in tumor volumes was found between the control group and other treatment groups.	Photodynamic therapy PET/CT validation		Duan et al. (2018) [37]
33	PEGylated crushed gold shell-radioactive iodide-124-labeled gold core nanoballs (PEG-124I-Au@AuCBs) C = 20 nm	124I	Bound	In vivo, in vitro, ex vivo Murine breast cancer (4T1)	PEG-124I-Au@AuCBs showed high stability and sensitivity in various pH solutions, serum, and in vivo conditions. Combined PET/CLI clearly revealed tumor lesions at 1 h after injection of particles, and both signals remained visible in tumor lesions at 24 h.	PET validation	IVIS Lumina III	Lee et al. (2018) [38]
34	Amorphous silica NP (SNP) H = 163.7 ± 4.8 nm TiO_2_ H = 12.8 ± 1.5 nm, HfO_2_ H = 34.9 ± 0.7 nm, Eu_2_O_3_ H = 134 ± 18 nm Gd_2_O_3_ H = 75.7 ± 6.8 nm YAG:Ce H = 36.6 ± 4.0 nm Bi2O3 H = 201.2 ± 4.4 nm AuNP H = 85.8 ± 0.009 nm	3H, 35S, 177Lu, 32P, 18F, 89Zr, 68Ga, 90Y 99mTc	Separated	In vitro, in vivo	β-scintillation contributes appreciably to excitation and reactivity in certain nanoparticle systems. The excitation by radionuclides of nanoparticles composed of large atomic number atoms generates X-rays, enabling multiplexed imaging through single photon emission computed tomography. Optical imaging and therapy using radionuclides with emission energies below the Cerenkov threshold are feasible, thereby expanding the list of applicable radionuclides.	SPECT/CT validation	IVIS Spectrum	Pratt et al. (2018) [25]
35	Magnetic nanoparticle(Zn_0.4_Mn_0.6_) Fe_2_O_4_ s (MNPs) with 89Zr radiolabeling and porphyrin molecules (TCPP) surface modification (89Zr-MNP/TCPP) C = 20 nm	89Zr	Bound	In vivo, in vitro, ex-vivo Murine breast cancer (4T1)	In vivo biodistribution of the 89Zr-MNP/TCPP imaging (FL), Cerenkov luminescence (CL) and CRET Imaging. High NPs tumor accumulation in the presence of an external magnetic field. The intensity spectrum of 89Zr-MNP/TCPP was completely different from that of free 89Zr and exhibited much stronger emission at a long wavelength, i.e., from 600 to 800 nm, reaching the maximum at around 660 nm.	Photodynamic therapy PET confirmation	IVIS system	Ni et al. (2018) [41]
36	Y_2_O_3_:Eu^3+^ rare earth nanoparticles (RENPS) C = 51.5 ± 7.5 nm	68Ga	Separated	In vitro.	Enhanced CL penetration and intensity (over 3 times better) by using Y_2_O_3_:Eu^3+^ RENPs. 3D reconstruction method which is able to acquire more accurate spatial information in vivo, as well as some quantitative information.	PET/CT validation	IVIS Kinetic	Gao et al. (2018) [59]
37	Gold nanoparticles C = 15 nm	Electron pulse	Separated	In vitro. No imaging	Cerenkov absorption spectrum in water with different gold nanoparticle concentrations			Ghandi et al. (2018) [60]
38	188Re-liposome H = 74.2 ± 9.1 nm	188Re	Bound	In vitro, in vivo, ex vivo Human hypopharyngeal carcinoma cells (FaDu) Human tongue carcinoma (SAS) Human oral squamous carcinoma (OECM-1)	CLI demonstrate an increased accumulation of 188Re-liposome in the tumor lesion of nude mice with repeated doses compared to a single dose.		IVIS 50	Chang et al. (2018) [28]
39	Radioiodine-labelled melanin nanoparticles (MNP–Ag–131I) C = 6 nm H = 12 nm	131I	Bound	In vitro, in vivo, ex vivo Human prostate carcinoma (PC3)	Synthesis of MNP–Ag–131I for therapeutic purposes which can be used for both single-photon emission computed tomography and Cherenkov radiation imaging. The beta rays of 131I make it a good candidate as a cancer cell killer.	MNP used as a platform for SPECT and CLI for accurate localization in brachytherapy.	IVIS Spectrum	Sheng et al. (2020) [61]
40	PEGylated crushed gold shell @ radioiodine-labeled core nanoparticles (PEG-124I-Au@AuCBs) C = 20 nm	124I	Bound	In vitro, in vivo, ex vivo.	PEG-124I-Au@AuCBs are promising lymphatic tracers for dual imaging PET/CLI. NPs allowed for high-sensitivity detection of SLNs within 1 h postinjection, and their accumulation persisted until 24 h in a clinical application under intraoperative conditions.	PET/CT validation	IVIS Lumina III	Lee et al. (2019) [22]
41	TiO_2_ nanoparticles coated with the glycoprotein transferrin (Tf) (Tf/TiO_2_) C = 25 ± 3.2 nm	18F	Separated	In vitro. No imaging Human multiple myeloma (MM1.S) and human fibrosarcoma (HT1080)	The use of an electrospray system is efficient for coating the protein transferrin (Tf) on the surface of titanium dioxide (TiO_2_) nanoparticles in a single step. Tf/TiO_2_ nanoparticles improved cell killing for MM1.S multiple myeloma cells from 23% to 57%, compared to Tf/TiO_2_ nanoparticles prepared using conventional functionalization methods.	Cerenkov Radiation Induced Cancer Therapy		Reed et al. (2019) [44]
42	TiO_2_ nanoparticles	18F	Separated	No imaging	Mathematic model that integrates Cerenkov physics, light interaction with matter, and photocatalytic reaction engineering. The model investigates the concentration of TiO_2_ nanoparticles and the activity of the radionuclide 18F-FDG on the number of photons and ROS generation. The model can be used for other radionuclides and nanoparticles, and can provide guidance on the concentration and size of TiO_2_ nanoparticles and the radionuclide activity needed for efficient cancer therapy.	Theoretical investigation Validation with comparison to experimental reports in the literature		Kavadiya et al. (2019) [62]
43	Europium nanoparticles Microspheres	X ray from LINAC	Separated		New methodological approach to reconstruct Cherenkov excited luminescence intensity distributions starting from a three-dimensional dataset. The method makes it possible to visualize and localize luminescence/fluorescence tagged vasculature, lymph nodes, or superficial tagged regions with most dynamic treatment plans.	Cherenkov excited luminescence scanning imaging (CELSI)	ICCD Princeton Instruments	Jia et al. (2019) [63]
44	Silica nanoparticles (Plus-NPs) H = 25 nm	32P	Separated	In vitro	Synthesized pluronic–silica nanoparticles doped with five different dyes that were chosen to efficiently absorb CR in all the visible ranges and to efficiently funnel the excitation energy toward the lowest energy dye, a Cy7 derivative, presenting a fluorescence emission in the near-infrared region (NIR).		IVIS Spectrum	Genovese et al. (2020) [26]
45	Chelate-Free Radiolabeling and Transferrin coating of TiO_2_ NPs (89Zr -TiO_2_- Tf NPs)	89Zr	Bound	In vitro, in vivo, ex vivo. Multiple myeloma (MM1.S)	Design of a theranostic nanoplatform (89Zr -TiO_2_-Tf NPs) for targeting bone marrow, imaging the distribution of NPs, and stimulating ROS generation for cell killing. Single dose of NPs inhibited cancer growth	Photodynamic therapy PET/CT imaging	IVIS Lumina	Tang et al. (2020) [64]
46	Persistent Luminescent ZnGa_2_O_4_:Cr^3+^, NPs (ZGCs) C = 60–80 nm	18F	Separated	In vitro, in vivo, ex vivo. Murine breast cancer (4T1)	18F can efficiently excite ZGCs Nanoparticles for both fluorescence and afterglow luminescence via Cerenkov resonance energy transfer, as well as ionizing radiation.	PET imaging	IVIS Lumina	Liu et al. (2020) 35]
